# The Remarkable Rise in High-Entropy Catalysts: A New Paradigm for Sustainable Hydrogen Production

**DOI:** 10.3390/nano16090548

**Published:** 2026-04-30

**Authors:** Abid Ahmad, Irshad Bhat, Qian Liu, Min Zhang, Sihao Lv, Faliang Cheng, Wei Li

**Affiliations:** 1 Research Center for Eco-Environmental Engineering, School of Environment and Civil Engineering, Dongguan University of Technology, Dongguan 523808, China; tawheedabid@dgut.edu.cn (A.A.);; 2 Guangdong Engineering and Technology Research Center for Advanced Nanomaterials, Dongguan University of Technology, Dongguan 523808, China; 3 Department of Physics, Government Degree College, Sopore 193201, Kashmir, India

**Keywords:** hydrogen evolution reaction (HER), green hydrogen production, high-entropy catalysts (HECs), active site mechanisms, operando characterization

## Abstract

The hydrogen evolution reaction (HER) is a cornerstone of green hydrogen production, yet its efficiency is constrained by the sluggish kinetics of water splitting. High-entropy catalysts (HECs), single-phase materials incorporating multiple principal elements, have emerged as a transformative solution. Their unique attributes including vast compositional flexibility, tunable electronic structures, and synergistic multi-element interactions, enable them to overcome the activity, stability, and cost limitations of conventional catalysts. Despite rapid performance advancements, the rational design of HECs is fundamentally hampered by critical knowledge gaps, particularly in identifying true active sites under operando conditions and predicting long-term stability. This work critically assesses these challenges, systematically summarizing the latest progress in HECs design, synthesis, and structure–activity relationships. By bridging fundamental principles with practical applications, we provide a forward-looking perspective on key research directions. Distinct from recent progress-focused reviews, this work establishes a strategic roadmap by systematically diagnosing seven grand challenges across the science-to-technology pipeline and proposing corresponding countermeasures. This framework aims to guide future research efforts toward the rational design and practical deployments of HECs for practical and cost-effective green hydrogen production.

## 1. Introduction

The escalating global energy demand and environmental degradation, driven by an overreliance on fossil fuels, necessitate a paradigm shift toward sustainable energy system. Hydrogen has emerged as a pivotal clean energy carrier, and its green production via electrochemical water splitting is a cornerstone of future energy landscapes. The efficiency of this process, however, is fundamentally limited by the sluggish kinetics of the hydrogen evolution reaction (HER), demanding advanced electrocatalysts to minimize overpotential $\left(\eta \right)$ and ensure long-term stability [1,2,3]. While platinum-group metals remain the benchmark for HER, their scarcity and high cost are prohibitive for global-scale deployment. Consequently, extensive research has focused on earth-abundant alternatives, yet conventional design strategies, typically confined to limited compositional spaces, often struggle to simultaneously optimize activity, durability, and cost. This impasse underscores the urgent need for novel catalyst design paradigms that transcend traditional materials discovery.

**Figure 1:**
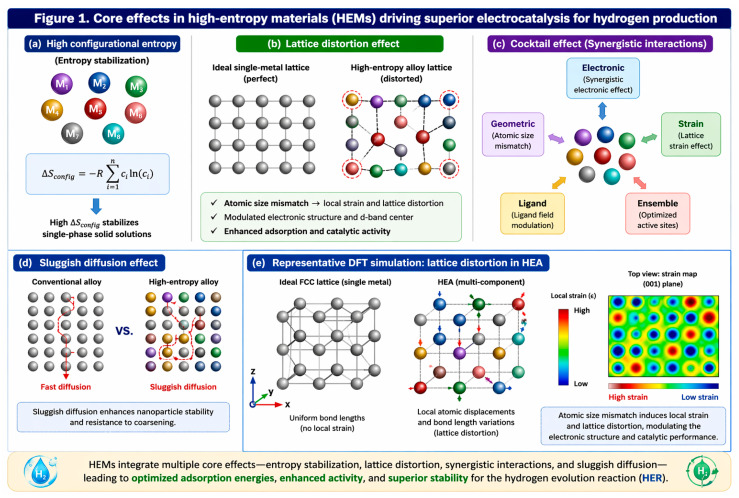
**Core effects in high-entropy materials (HEMs) relevant to HER catalysis:** (**a**) configurational entropy stabilization, (**b**) lattice distortion effect arising from atomic size mismatch, (**c**) cocktail effect and synergistic multi-element interactions, (**d**) sluggish diffusion effect, and (**e**) representative DFT-inspired schematic illustrating local atomic displacement, bond-length variation, and strain distribution in a high-entropy alloy (HEA), based on reported theoretical studies.

In this context, high-entropy materials (HEMs) [4,5,6,7,8] have emerged as a promising platform for catalyst design [9]. The compound of multicomponent, entropy-stabilized materials was first established in 2004 through the pioneering works of Yeh et al. and Cantor et al., which laid the foundation for modern high-entropy systems [10,11]. Rather than being defined by a strict number of elements, high-entropy materials are more broadly understood as a multicomponent system in which configurational entropy plays a dominant role in stabilizing single-phase structures, even when the number of constituents elements varies. This entropy-driven stabilization (Δ*S_mix_*) enables the formation of structurally robust solid solutions with unique physicochemical properties [12]. As illustrated in Figure 1a–d, such systems exhibit several key features beneficial for catalysis. Entropy stabilization helps maintain phase integrity under operating conditions, lattice distortion introduces a wide distribution of active sites and defect structures, and multi-element electronic interactions enable fine tuning of adsorption energetics (ΔG_H*_). Reported DFT studies have shown that atomic size mismatch in high-entropy alloys can induce local atomic displacements, bond-length variations, and strain fields, which strongly influence the surface electronic structure and adsorption energetics. A representative schematic based on these theoretical insights is presented in Figure 1e to better visualize the lattice distortion effect [13,14]. Collectively, these three effects of entropy stabilization, lattice distortion, and electronic tuning form the fundamental triad that governs the unique activity and durability of HEM-based catalysts for HER [15,16], although the same structural complexity can also introduce stability challenges such as phase segregation or surface restructuring under operation.

The unique behavior of high-entropy materials is generally understood through several interconnected core effects. The cocktail effect refers to the synergistic interaction among multiple elements, which can create catalytic properties beyond those of simpler systems. Lattice distortion results from differences in atomic size and bonding environments, generating local strain that can tune adsorption energetics and defect formation. Sluggish diffusion arises from the complex atomic landscape and can suppress elemental segregation while improving structural stability during operation. Together, these effects provide a systematic framework for understanding the catalytic activity and durability of high-entropy catalysts.

The theoretical promise of high-entropy catalysts (HECs) has been rapidly substantiated by a growing body of experimental work. For instance, high-entropy alloys (HEAs) that strategically incorporate trace amounts of platinum (Pt) with several non-precious metals have demonstrated Pt-like HER activity at a fraction of the cost, effectively decoupling performance from price [17]. Similarly, high-entropy oxides (HEOs) such as (CoCrFeNiMn)_3_O_4_ exhibit exceptional durability and high activity, driven by their multi-cationic structures that optimize charge transfer kinetics and resist corrosion [18]. Crucially, the intrinsic stability endowed by the high-entropy effect has been shown to withstand industrial-scale current densities (>500 mA cm^−2^), directly addressing a critical bottleneck for practical electrolyzer technologies [19].

Despite these promising advances, several critical challenges remain that limit the practical implementation of high-entropy catalysts. The complex multicomponent compositions that give rise to entropy stabilization can also introduce issues such as phase segregation, local compositional fluctuations, and surface reconstruction during electrochemical operation. These phenomena may lead to performance degradation, particularly under high current densities or prolonged operation. Furthermore, the vast compositional design space of high-entropy systems makes it difficult to precisely control active site distributions and establish clear structure–activity relationships. Importantly, some of these limitations originate from intrinsic thermodynamic and kinetic complexities of multicomponent materials, whereas others are strongly dependent on synthesis strategies, elemental miscibility, and processing conditions. Therefore, understanding how these factors influence catalytic performance is essential for guiding rational catalyst design.

The rapid growth of HECs for HER, together with increasing interest in scalable green hydrogen production, makes a timely critical review of this field particularly important. The recent studies reported further demonstrate the accelerating progress of high-entropy electrocatalysts, highlighting continued advances in catalytic activity, structural tunability, and long-term durability [20,21,22]. Although several recent review articles have discussed high-entropy materials in broader electrocatalytic or energy-related applications, a focused review dedicated to HER remains comparatively limited. In particular, a clear framework connecting fundamental high-entropy effects, synthesis strategies, mechanistic understanding, performance trends, and practical challenges has not been systematically established. This review aims to address that gap by providing an integrated perspective on the design principles, catalytic mechanisms, current limitations, and future opportunities of high-entropy catalysts for HER.

## 2. Fundamentals of HER Electrocatalysis and HECs

### 2.1. Foundational Principles of HER

The HER represents a cornerstone of electrochemical energy conversion, where efficient hydrogen production relies on the delicate balance between proton–electron transfer and hydrogen adsorption energy. In the context of high-entropy catalysts (HECs), this balance is no longer governed by a single, uniform active site but by an ensemble of chemically distinct surface environments, each contributing differently to HER kinetics. Consequently, the overall HER process is dictated not only by the availability of optimal binding sites, but also by the statistical distribution of adsorption energies and local electronic structures intrinsic to high-entropy surfaces, which can mitigate under different pH conditions.

#### 2.1.1. Reaction Pathways

**Figure 2:**
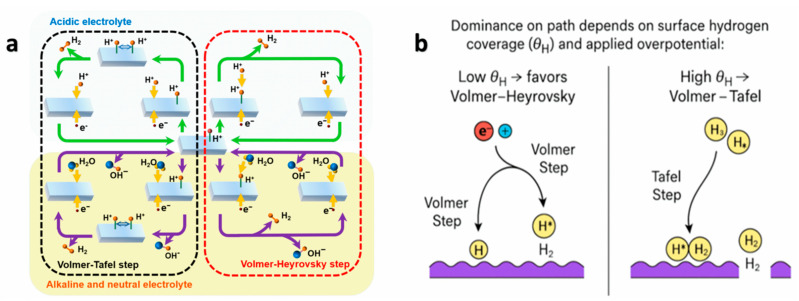
**Mechanistic pathways of the HER under acidic and alkaline conditions.** (**a**) Schematic illustration of the Volmer–Heyrovsky–Tafel mechanisms under different pH environments. In acidic media, protons (H^+^) are directly reduced to adsorbed hydrogen (H*) via the Volmer step, followed by either electrochemical desorption (Heyrovsky step) or chemical recombination (Tafel step) to form H_2_. In alkaline media, water dissociation provides the hydrogen source, generating H* and OH^−^ intermediates through the Volmer step, with subsequent Heyrovsky or Tafel coupling to evolve H_2_. (**b**) Pathway dominance depends on surface hydrogen coverage $\theta_{H}$ and applied overpotential: at low $\theta_{H}$, the Volmer–Heyrovsky route dominates, whereas at high ($\theta_{H}$), the Volmer–Tafel pathway prevails.

In acidic electrolytes, protons are reduced, while in alkaline media water dissociation supplies the protons required for H_2_ formation. The sequence begins with the generation of surface-adsorbed hydrogen intermediates (H* or H_ad_), which then combine or react with additional protons/electrons to evolve hydrogen molecules. On HEC surfaces, the formation and stabilization of H* species occur on chemically non-equivalent metal sites, enabling parallel reaction pathways with locally optimized energetics. Depending on the surface characteristics and applied potential, two primary routes are involved: the Volmer–Heyrovsky and Volmer–Tafel mechanisms [23,24,25,26]. A schematic overview of these mechanisms under both pH conditions is presented in Figure 2a.

1. Volmer–Heyrovsky pathway (electrochemical desorption route):

Volmer step (discharge):

H_3_O^+^ + e^−^ → H* + H_2_O (in acidic media) (1)

H_2_O + e^−^ → H* + OH^−^ (in alkaline media) (2)

Heyrovsky step (electrochemical desorption):

H* + H_3_O^+^ + e^−^ → H_2_ + H_2_O (acidic) (3)

H* + H_2_O + e^−^ → H_2_ + OH^−^ (alkaline) (4)

2. Volmer–Tafel pathway (chemical desorption route):

Volmer step (discharge):

H_3_O^+^ + e^−^ → H* + H_2_O (acidic) (5)

H_2_O + e^−^ → H* + OH^−^ (alkaline) (6)

Tafel step (chemical recombination):

H* + H* → H_2_ (7)

The preferred pathway is governed by the surface hydrogen coverage $\left(\theta_{H}\right)$ and the applied overpotential $\left(\eta \right)$ (Figure 2b). At relatively low $\;\theta_{H}$, the Volmer–Heyrovsky pathway dominates, reflecting the facile desorption of single H* species. In HECs, this regime is enhanced by the presence of weak to moderate hydrogen-binding sites, which accelerate electrochemical desorption. At higher $\;\theta_{H}$, the Volmer–Tafel pathway becomes favorable, as increased surface coverage promotes recombination of adjacent H* species. Importantly, the spatial proximity of diverse metal atoms in HECs facilitates H* migration between sites, lowering recombination barriers and broadening the operational window for the Tafel step [27,28]. This interplay underscores the critical role of adsorption energetics, site diversity, and surface interactions in dictating HER kinetics on high-entropy surfaces.

To further elucidate the reaction mechanisms in high-entropy catalysts, operando and in situ characterization techniques have become increasingly important. Techniques such as X-ray absorption spectroscopy (XAS), including XANES and EXAFS, provide direct insight into the evolution of local electronic structure and coordination environments under working conditions. In parallel, in situ Raman spectroscopy enables the identification of reaction intermediates and dynamic bonding changes during HER. These approaches are particularly relevant for high-entropy systems, where multiple elements contribute synergistically and surface reconstruction may occur during operation. Consequently, operando studies offer critical evidence for identifying active sites and establishing reliable structure–activity relationships in these complex catalytic systems [29,30].

#### 2.1.2. Key Performance Metrics

**Figure 3:**
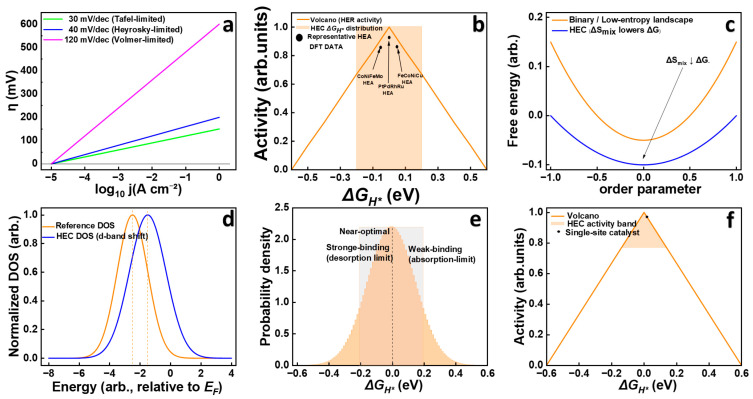
**Key electrochemical descriptors and theoretical relationships for evaluating HER performance of high-entropy catalysts:** (**a**) Tafel slope analysis, (**b**) HER volcano plot with representative HEA DFT data, (**c**) entropy-stabilized free-energy landscape, (**d**) d-band center shift and DOS modulation, (**e**) distribution of hydrogen adsorption energies on heterogeneous HEA sites, and (**f**) comparison of activity windows for single-site and HEA catalysts.

The HER is conventionally evaluated using three fundamental electrochemical descriptors: overpotential $\left(\eta \right)$, exchange current density $\;(j_{0})$, and the Tafel slope (*b*). For HECs, these descriptors reflect the collective response of multiple active sites rather than a single dominant catalytic center, providing a more holistic measure of catalytic performance. Figure 3 summarizes the key electrochemical descriptors and theoretical relationships commonly used to evaluate HER performance in high-entropy catalysts, including Tafel analysis, volcano trends, adsorption energetics, and electronic structure effects. In addition, representative HEA computational data based on reported DFT hydrogen adsorption energies have been incorporated into Figure 3b to better contextualize the volcano relationship for multicomponent catalysts. These results indicate that HEA surfaces can tune ΔG_H*_ values toward the near-optimal binding region for enhanced HER activity [13].

Overpotential $\left(\eta \right)$ denotes the additional potential beyond the thermodynamic equilibrium value required to sustain a given current density. It originates from three principal contributions: (i) *activation losses*, associated with charge transfer barriers at the catalyst electrolyte interface; (ii) *ohmic losses*, resulting from internal resistances within the electrode, membrane, and electrolyte; (iii) *concentration losses*, caused by mass transport limitations at elevated current densities [31]. In HECs, the coexistence of sites with varying activation barriers can reduce the effective activation overpotential by enabling the reaction to proceed preferentially through the most energetically favorable pathways. Consequently, the value of η measured at 10 mA cm^−2^ $\left(\eta_{10}\right)$ is widely adopted as a benchmarking metric under technologically relevant conditions.

Exchange current density $\left(j_{0}\right)$ quantifies the intrinsic reaction rate at equilibrium, representing the rate at which forward and reverse HERs balance. A higher $j_{0}$ signifies a more active surface capable of facilitating rapid charge transfer and proton reduction. In HECs, elevated $j_{0}$ values often arise from the synergistic interaction of multiple metallic species, which enhances electronic conductivity and increases the density of catalytically accessible sites.

The Tafel slope (*b*), derived from the relationship between η-log j plots (Figure 3a), provides mechanistic insights by indicating the rate-determining step (RDS). Classical values of ~120, ~40, and ~30 mV dec^−1^ correspond to the Volmer, Heyrovsky, and Tafel steps, respectively. However HECs frequently exhibit intermediate slopes (30–120 mV dec^−1^), reflecting mixed reactions pathways occurring simultaneously on chemically distinct sites, as well as contribution from surface heterogeneity and dynamic restructuring under operating conditions [32,33].

Therefore, an ideal HER catalyst is characterized by a low η for energy efficiency, a high $j_{0}$ for intrinsic activity, and an optimal *b* reflecting favorable reaction kinetics. In HECs these metrics collectively define the ensemble averaged catalytic behavior, rather than the performance of a single active site.

#### 2.1.3. The Sabatier Principle and Activity Descriptors

The Sabatier principle states that optimal catalytic activity occurs when reaction intermediates are bound neither too strongly nor too weakly. For HER, the governing descriptor is the hydrogen adsorption free energy (ΔG_H*_). When ΔG_H*_ ≪ 0, hydrogen overbinds and desorption is hindered; when ΔG_H*_ ≫ 0, adsorption becomes rate-limiting. Peak activity resides near ΔG_H*_ ≈ 0 eV, a balance traditionally embodied by Pt.

While this principle provides a foundational theoretical framework for evaluating and designing HER catalysts, the assumption of a single, uniform adsorption energy is an idealization. In practice, catalyst surfaces exhibit heterogeneous adsorption environments arising from structural defects, local coordination variations, and compositional diversity. This heterogeneity is intentionally amplified in HECs, which present a broad distribution of active sites with varying ΔG_H*_ values. Rather than relying on a single optimal adsorption site, these catalysts operate through a spectrum of local atomic configurations that collectively enable efficient hydrogen adsorption and desorption (Figure 3b). As discussed in Section 2.2, this ensemble site behavior allows different surface environments to facilitate distinct elementary steps of the HER, thereby broadening the activity window while maintaining catalytic robustness across varying electrochemical conditions.

### 2.2. Core Principles of HECs

The catalytic behavior of high-entropy catalysts arises from two distinct but complementary mechanisms: thermodynamic stabilization and electronic/kinetic synergistic effects. Thermodynamic effects are primarily governed by configurational entropy Δ*S_mix_*, which lowers the Gibbs free energy and stabilizes single-phase solid solutions against segregation. This entropy-driven stabilization helps suppress phase segregation and maintain structural integrity under electrochemical operating conditions. In contrast, electronic and kinetic effects originate from synergistic interactions among multiple constituent elements, often referred to as the “cocktail effect”. These interactions modify the local electronic structure, shift the d-band center, and tune the hydrogen adsorption free energy ΔG_H*_, thereby influencing the reaction kinetics of HER. As schematically illustrated in Figure 3c,d, entropy-driven thermodynamic effects primarily govern structural stability, whereas electronic and synergistic interactions determine catalytic activity and reaction kinetics [34,35]. Distinguishing these two contributions provides a clearer conceptual framework for understanding the design principles of high-entropy electrocatalysts.

Rapid nanoscale syntheses such as flash pyrolysis, solvothermal routes, and carbothermal shock help lock in uniform, high-surface-area structures, while computational approaches (DFT, high-throughput screening, machine learning) guide the optimization of ΔG_H*_, DOS, and charge distribution [36]. The following sections further discuss how these core effects influence catalytic behavior and active site chemistry.

#### 2.2.1. The High-Entropy Concept: A Paradigm Shift in Materials Design

Entropy-driven stabilization provides the thermodynamic foundation of HECs. A high Δ*S_mix_* lowers Δ*G* (Δ*G* = Δ*H* − *T*Δ*S*) and suppresses phase segregation, while sluggish diffusion further contributes to structural retention during operation. Structural disorder manifested as lattice distortion, amorphous domains, or vacancy-rich regions broadens the adsorption site spectrum and introduces coordinatively unsaturated sites that accelerate kinetics. Experimental demonstrations span porous single-phase HEOs/HESs robust in water-splitting conditions and high-entropy double perovskites exhibiting low η with sustained stability [37,38,39,40]. These entropy-driven effects therefore mainly influence the thermodynamic stability of high-entropy materials by suppressing phase segregation and maintaining single-phase structures during catalytic operation, as conceptually illustrated in Figure 3c. Collectively, these effects underpin the long-term resilience of HECs relative to Pt- and Pd-based benchmarks, especially under harsh electrolytes and extended operation.

#### 2.2.2. The “Cocktail Effect”: Tailoring Electronic and Geometric Structure

Multi-element synergistic interactions (cocktail effect) strongly influence both the geometric and electronic structure of catalysts. Lattice distortion and local strain adjust bond lengths and orbital overlap, while multi-metal coordination alters d-band centers and redistributes electron density. These interlinked perturbations collectively tune ΔG_H*_ toward the Sabatier window, not as a single fixed value but as a distribution, which enhances tolerance to surface evolution and environmental variability. Unlike entropy-driven thermodynamic stabilization, the cocktail effect primarily governs the electronic structure and catalytic kinetics of high-entropy catalysts by tuning adsorption energetics and lowering reaction barriers for HER, as schematically shown in Figure 3d [14,41,42,43,44,45]. Such regulation directly explains the experimentally observed low η. For instance, noble metal-containing HEAs such as PtPdRuCuNi and high-entropy metallic glasses like AlMnYNiCoAu exhibit η < 40 mV at 10 mA cm^−2^ in alkaline media, while CoCrFeNi alloys attain ≈60 mV at 1 mA cm^−2^ in acidic media due to stabilization of Ni-rich surfaces [46,47,48,49].

These results exemplify how cooperative electronic coupling and strain modulation within multimetallic matrices can effectively reduce activation barriers, optimize water dissociation, and achieve platinum-like HER performance across both acidic and basic environments.

#### 2.2.3. Redefining the Active Site: Beyond the Single-Site Model

The core mechanistic paradigm of high-entropy catalysis is the division of labor between strong- and weak-binding sites. On the heterogeneous surfaces of HECs, strong-binding microenvironments facilitate hydrogen adsorption during the Volmer step, while weak-binding domains accelerate hydrogen desorption via Heyrovsky or Tafel steps. This cooperative interplay—schematically depicted by the spatial division of labor in Figure 3e,f—creates a self-regulating reaction network in which different local atomic configurations specialize in distinct roles. The outcome is minimized activation loss and sustained charge transfer efficiency across broad potential and pH ranges.

Such ensemble behavior departs fundamentally from the uniform, single-site models of conventional catalysts. The coexistence of diverse adsorption energetics leads to optimized reaction kinetics, producing high exchange current densities $j_{0}$ and intermediate *b* (~50–60 mV dec^−1^), reflecting mixed or coverage-dependent rate-determining steps [36,50,51]. Porous and amorphous multicomponent alloys further exemplify this principle by offering a continuous spectrum of adsorption and desorption microenvironments, thereby elevating turnover frequency without reliance on uniform Pt-like motifs.

Moreover, the inherent ΔG_H*_ distribution buffers HER activity against structural evolution, poisoning, or pH variation, ensuring stable and nearly pH-universal operation. What was once regarded as structural or electronic disorder thus becomes a catalytic advantage—transforming compositional heterogeneity into a functional design principle that yields surfaces that are both highly active and intrinsically adaptive under electrochemical conditions.

#### 2.2.4. Entropy Engineering vs. Traditional Systems: Advantages of HECs

Compared with conventional single-metal or binary catalysts, high-entropy catalysts exhibit a fundamentally different catalytic paradigm. Their activity and stability arise from the combined influence of entropy-driven structural stabilization, multi-element electronic interactions, and ensemble active site behavior described in Section 2.2.1, Section 2.2.2 and Section 2.2.3. These mechanisms collectively generate a distribution of hydrogen adsorption energies across the catalyst surface, enabling efficient hydrogen adsorption, migration, and desorption during HER. As a result, HECs frequently demonstrate low overpotentials η, high exchange current density $\;j_{0}$ and favorable reaction kinetics across a broad range of pH conditions. A comparison between HECs and traditional catalyst systems is summarized in Table 1. It should be noted that Table 1 provides a general comparison of key characteristics and representative performance ranges. The reported values are collected from different studies under varying experimental conditions and are intended to illustrate trends rather than provide direct benchmarking.

### 2.3. Synthesis of HECs

**Figure 4:**
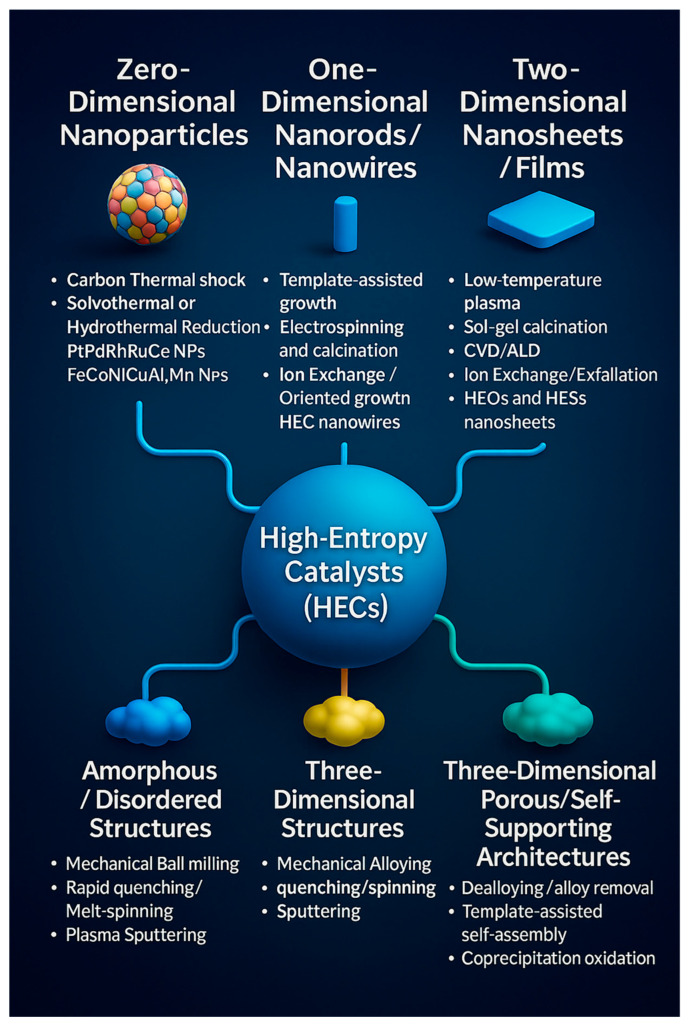
**Classification of synthetic strategies for HECs based on structural dimensionality.** The schematic categorizes HEC synthesis routes according to the morphology of the resulting products: 0D nanoparticles, 1D nanorods/nanowires, 2D nanosheets or thin films, 3D porous or self-supporting architectures, and amorphous/disordered structures. Representative synthesis approaches include carbon thermal shock, solvothermal reduction, electrospinning, plasma-assisted processing, template-directed assembly, and mechanical ball milling. This classification highlights the direct relationship between synthetic strategy and resulting structural dimensionality, offering a rational framework for catalyst design optimization and structure–activity correlation in high-entropy systems [56].

The synthesis of HECs can be systematically classified according to their structural dimensionality, encompassing zero-dimensional (0D) nanoparticles, one-dimensional (1D) nanowires, two-dimensional (2D) nanosheets, three-dimensional (3D) porous architectures, and amorphous frameworks. Each dimensional class arises from distinct synthesis methodologies that determine not only morphology but also catalytic behavior in the HER. This framework bridges synthesis strategy and structural outcome, offering a rational foundation for optimizing HER performance through controlled dimensional design (Figure 4).

Zero-dimensional (0D) nanoparticles are typically produced through nonequilibrium, rapid-heating routes such as carbothermal shock (CTS), solvothermal, or hydrothermal reduction. These techniques yield uniformly mixed, single-phase HEA or HEO nanoparticles—for example, PtPdRhRuCe/C and FeCoNiCuMo–O—with high surface areas and catalytic stability [52,53]. Such nanostructures maximize atomic dispersion and active site exposure, essential for efficient HER.

One-dimensional (1D) nanorods and nanowires, fabricated by template-assisted growth, electrospinning, or ion exchange-oriented synthesis, offer direct electron transfer pathways and reduced gas diffusion resistance. Their elongated morphology facilitates charge transport and structural robustness, as demonstrated in template-grown HEC nanowires and nanorods [54,55].

Two-dimensional (2D) nanosheets and thin films are obtained via low-temperature plasma, sol–gel calcination, or atomic-layer deposition (ALD). Their ultrathin nature exposes abundant defect sites and promotes rapid ion migration. HEO and HES nanosheets prepared by exfoliation or ion exchange exhibit high surface defect density and enhanced conductivity, leading to superior catalytic activity [57].

Three-dimensional (3D) structures, including both dense alloys and porous/self-supporting architectures, are synthesized through mechanical alloying (MA), rapid quenching, sputtering, or dealloying/self-assembly techniques. For instance, FeNiCoCuMo and CoCrFeNi HEAs synthesized by MA maintain single-phase stability and electrical connectivity under industrial-scale HER conditions [52]. In parallel, template-assisted self-assembly or dealloying methods create 3D porous frameworks that combine mechanical integrity with high active site accessibility.

Amorphous or disordered HECs, formed via ball milling, plasma sputtering, or rapid-quenching routes, feature locally distorted atomic arrangements that generate a spectrum of adsorption energies (ΔG_H*_). This intrinsic heterogeneity endows them with flexible catalytic behavior and long-term durability under variable operating conditions.

Collectively, these synthesis strategies—summarized in Figure 4 and detailed in Table 2—demonstrate that dimensional control and synthesis route are inherently interlinked: 0D–2D morphologies maximize surface reactivity and charge transport, while 3D and amorphous systems provide enhanced mechanical robustness and mass transport. However, achieving scalable, contamination-free fabrication and developing operando phase-monitoring tools remain central challenges. The synergy between dimensional design and method-guided synthesis thus represents a critical frontier for advancing HECs toward industrially viable, sustainable hydrogen production technologies.

In addition to synthesis scalability, the practical deployment of high-entropy catalysts also depends on raw material cost, elemental sustainability, and processing complexity. While Pt-based catalysts remain the benchmark for acidic electrolyzers and Ni-based catalysts are widely used in alkaline systems, the economic viability of HECs is strongly composition-dependent. Compositions containing critical raw materials such as Co or noble metals may face cost constraints, whereas earth-abundant multicomponent systems could provide a more competitive route for future large-scale applications.

## 3. Progress in HECs for HER

**Figure 5:**
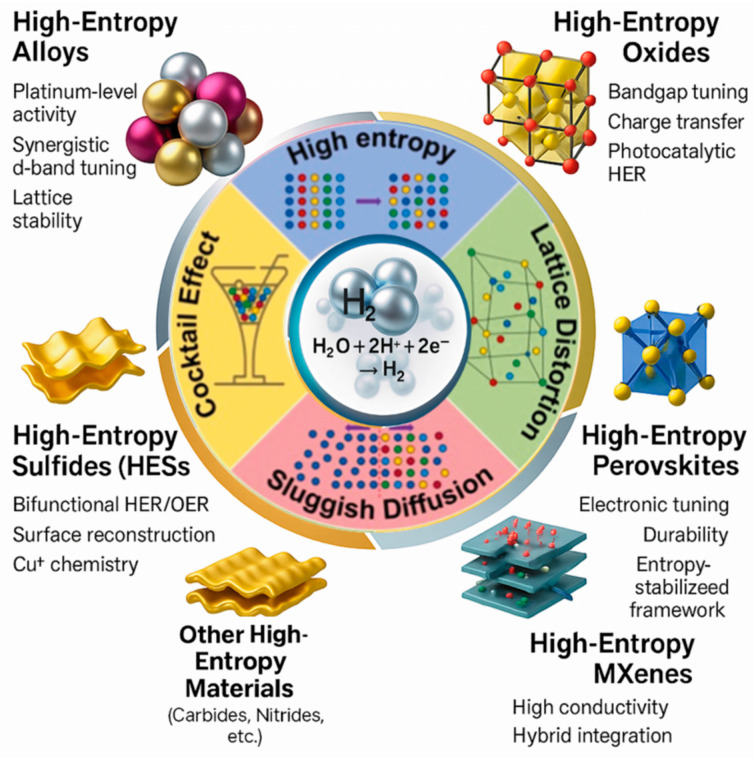
**Overview of progress in high-entropy catalysts (HECs) for the hydrogen evolution reaction (HER).** Schematic illustration summarizing the evolution of high-entropy catalyst classes and their governing core effects. The central HER process (H_2_O + 2H^+^ + 2e^−^ → H_2_) is surrounded by the four fundamental high-entropy effects—cocktail effect, high-entropy effect, lattice distortion, and sluggish diffusion—that collectively enhance catalytic activity, stability, and universality. Representative material families are shown: HEAs exhibiting platinum-level activity and synergistic d-band tuning; HEOs with bandgap tuning and charge transfer capability; HESs featuring bifunctional HER/OER performance and Cu^+^-mediated surface chemistry; HEPs offering electronic tunability and structural durability; high-entropy MXenes with high conductivity and hybrid integration potential and other high-entropy materials [60].

Having established the fundamental principles of HER and the unique theoretical advantages of HECs, we now turn to the experimental breakthroughs that have solidified their status as transformative platforms for catalysis. This section summarizes the development of different HEC material classes from the pioneering high-entropy alloys to oxides, sulfides, and emerging two-dimensional systems and highlights the key structural features responsible for their catalytic performance. Figure 5 provides a visual overview of the material systems discussed in this section.

### 3.1. High-Entropy Alloys (HEAs): The Pioneering Platform

HEAs were the first to demonstrate the potential of entropy-driven design in electrocatalysis and remain the benchmark for HER performance. Platinum-containing HEAs such as PtPdRhRuCu nanoparticles exhibit η as low as ~23 mV at 10 mA cm^−2^ in alkaline solution [46], outperforming Pt/C. Ultra-small Pt(FeCoCuNi) nanocrystals further lower η to ~20 mV, achieving mass activities near 72 A mg^−1^ Pt with stability beyond 50 h [61]. Likewise, NiCoFePtRh HEAs maintain mass activities of ~28.3 A mg^−1^ Pt in acidic HER, retaining stability through 10,000 cycles [62]. These results highlight the ability of HEAs to combine platinum-level activity with superior durability. PGM-free HEAs are equally impressive. For instance, FeCoNiCuAl_2_Mn prepared via dealloying delivers $\eta_{10}\;$ of 9.7 mV in 1 M KOH and retains activity for over 100 h [63]. CoCrFeNi alloys achieve η of 107 mV in acid and 172 mV in base, with corrosion resistance comparable to Pt sheets [64].

The remarkable performance of these HEAs is not accidental; it stems directly from the synergistic interplay of their constituent elements. Mechanistic studies attribute these performances to synergistic interactions among multiple elements. Oxidized Ni and Co accelerate water dissociation, while Pt or Pd sites optimize hydrogen adsorption and recombination [48,65]. In FeCoPdIrPt, Fe and Co donate electrons to Pt, lowering its d-band center and reducing η to ~42 mV [66]. Mo and Zn incorporation in FeCoNi reduces barriers for water activation, providing dual activation [67]. Even otherwise inactive Cu sites can be activated through charge redistribution, as shown in FeCoNiCuMn, which achieves bifunctional HER/OER performance [68]. Structurally, lattice distortion and sluggish diffusion maintain single-phase stability and prevent segregation during operation [69].

In direct comparison, Pt/C performs well in acidic HER but degrades in alkaline media and remains costly. MoS_2_, though inexpensive, suffers from higher η (74–126 mV) and requires heterostructure to achieve multifunctionality [47]. HEAs uniquely integrate platinum-level activity, broad pH universality, and scalable synthetic routes, establishing themselves as the pioneering platform for high-entropy electrocatalysis.

### 3.2. Expanding the Horizon: High-Entropy Oxides (HEOs), Sulfides (HESs) and Beyond

To further reduce reliance on noble metals and broaden functional diversity, the high-entropy design concept has been extended to oxides and sulfides. These non-metallic systems introduce new catalytic mechanisms, such as dynamic surface self-reconstruction, tailored surface chemistry, nanostructure multiplicity, and even photocatalytic activity, yielding highly versatile and durable HER catalysts. For example, layered sulfides such as (Fe_0.2_Mn_0.2_Ni_0.2_Co_0.2_Mo_0.2_)S_2_ display increased surface area and active site density, achieving highly efficient alkaline HER kinetics with low η and robust stability [70]. Amorphous CuCoNiMnCrS_x_ nanosheets grown on Ni foam show ultra-low onset potentials (~0.25 V) and sustain current densities up to 1 A cm^−2^ for over 100 h, aided by Cu^+^-mediated “soft acid–hard acid” surface chemistry [38]. Entropy-stabilized sulfides such as (ZnCoMnFeAlMg)_9_S_8_ provide multifunctionality, achieving $\eta_{10}$ ≈ 170 mV for HER while excelling in OER [71].

High-entropy oxides expand the design space by enabling photocatalytic HER. TiHfZrNbTaO_11_, featuring dual perovskite/orthorhombic phases and a bandgap of ~2.9 eV, drives platinum-free HER under visible light [72]. Electronegativity contrast within such oxides enhances water adsorption and charge transfer [73].

Four mechanistic advances characterize these systems. Self-reconstruction processes convert Ru-bearing sulfides into active oxyhydroxide surfaces during operation [74], while surface chemistry optimization, particularly Cu^+^ environments, accelerates intermediate binding [38]. Nanostructure multiplicity in entropy-stabilized frameworks enhances site exposure and bifunctionality [75]. Finally, entropy-driven bandgap tuning enables visible-light HER in photocatalytic oxides [76]. Collectively, HEOs and HESs represent scalable, noble metal-free HER catalysts optimized for large-current operation and bifunctional electrolyzers.

### 3.3. Emerging Frontiers: High-Entropy Perovskites and MXenes

High-entropy perovskites (HEPs) represent a significant frontier, applying engineering to the already versatile perovskite oxide framework. This strategy yields catalysts with exceptional structural stability and tunable electronic properties. La_2_(Co_1/6_Ni_1/6_Mg_1/6_Zn_1/6_Na_1/6_Li_1/6_)RuO_6_ achieves $\eta_{10}$ ≈ 40.7 mV with stability exceeding 80 h in 1 M KOH, benefiting from alkali-induced super-exchange interactions that promote electron transfer [39]. Similarly, La(CrMnFeCoNi)O_3_ with electron filling near 1.2 provides a continuum of active sites [77]. A-site substitutions such as Sr doping further enhance charge transfer, enabling stable water splitting for 200 h [78]. Though still early in development, HEPs showcase how entropy-driven lattice and electronic tuning can yield platinum-free HER activity with extended durability [79].

High-entropy MXenes represent another frontier, derived from multi-principal MAX precursors. TiVNbMoC_3_ with oxygen terminations approaches thermoneutral ΔG_H*_ (~−0.41 eV), outperforming conventional ternary MXenes [80]. Their enlarged interlayer spacings (~1.5–1.8 nm) and high surface areas (~28 m^2^ g^−1^) enhance ion transport and site accessibility [81]. Morphological features such as crumpling and porosity mitigate restacking, while –O/–OH surface terminations further improve HER kinetics [82]. MXenes also integrate effectively into hybrids such as Ti_3_C_2_–TiO_2_ photocatalysts and support single-atom catalysts, broadening their application [83]. By combining high conductivity, tunable chemistry, and structural disorder, high-entropy MXenes emerge as scalable, platinum-free 2D platforms for HER.

### 3.4. Summary and Comparative Assessment

Across HEAs, HEOs/HESs, HEPs, and MXenes, high-entropy design strategies have created a diverse family of catalysts with tunable electronic structures, compositional flexibility, and improved catalytic stability for the HER. However, the catalytic performance, stability, and practical applicability of these materials vary significantly across different catalyst classes. To facilitate a clearer comparison of reported systems, a comparative overview of representative high-entropy catalyst classes for HER, including their catalytic mechanisms, performance metrics, durability, advantages, and practical limitations, is summarized in Table 3. This comparative overview enables a more systematic evaluation of the strengths and limitations associated with each class of high-entropy catalysts.

From this comparison, several important trends can be identified. HEAs generally exhibit the highest catalytic activity, often achieving very low overpotentials and broad pH applicability. This performance originates from strong electronic interactions among constituent elements, which enable fine tuning of hydrogen adsorption energetics and facilitate efficient reaction pathways. In contrast, high-entropy oxides and sulfides (HEOs/HESs) provide promising noble metal-free alternatives with enhanced structural robustness and corrosion resistance. Their catalytic activity is frequently influenced by dynamic surface reconstruction processes, which can generate highly active oxyhydroxide or defect-rich surface structures during operation.

Emerging catalyst systems such as high-entropy perovskites (HEPs) and MXenes further expand the design space for HER electrocatalysis. High-entropy perovskites offer highly tunable electronic structures through A-site and B-site compositional engineering, enabling control over charge transfer processes and catalytic intermediates. Meanwhile, high-entropy MXenes combine high electrical conductivity with tunable surface terminations and expanded interlayer spacing, providing favorable conditions for hydrogen adsorption and rapid electron transport. Although these emerging systems show promising catalytic behavior, they remain at relatively early stages of development and require further investigation to fully understand their catalytic mechanisms and long-term stability.

Despite the remarkable catalytic activity reported for many high-entropy catalysts, several practical challenges remain before these materials can be translated into large-scale hydrogen production technologies. First, some of the most active HEAs still rely on noble metals such as Pt, Pd, or Ru, which increases catalyst cost and limits economic scalability. Second, achieving uniform elemental distribution in multicomponent systems remains challenging during synthesis, particularly when scaling up production while maintaining precise compositional control. Third, many reported catalysts are evaluated under laboratory conditions and relatively short stability tests, which may not fully represent the demanding environments encountered in industrial electrolyzers.

Addressing these limitations will require the development of scalable synthesis strategies, standardized testing protocols, and long-term durability studies under industrially relevant current densities. Future research should therefore focus not only on maximizing catalytic activity but also on improving structural stability, reducing material cost, and ensuring reliable performance under realistic operating conditions.

### 3.5. Dynamics Surface Evolution and Lattice Oxygen Effects HECs

In addition to compositional complexity, many high-entropy catalysts undergo dynamic structural changes during electrochemical operation. Surface reconstruction, including elemental redistribution, changes in oxidation state, and the formation of hydroxylated or disordered surface layers, can significantly alter the nature of the active sites. As a result, the catalytically active phase may differ from the as-prepared material, making operando characterization especially important. For oxide-based high-entropy systems, lattice oxygen may also participate in interfacial bond rearrangement and intermediate stabilization under certain conditions. The extent of this contribution depends on factors such as metal–oxygen bonding strength, local coordination environment, and applied potential. Therefore, surface reconstruction, active phase evolution, and lattice oxygen participation should be considered as interconnected processes when evaluating the catalytic behavior of high-entropy systems.

## 4. Grand Challenges and Strategic Roadmap for High-Entropy HER Catalysis

While the progress discussed in the previous sections demonstrates the remarkable potential of high-entropy catalysts’ HER, translating these materials from laboratory discoveries into practical hydrogen production technologies requires addressing several critical challenges. Beyond catalytic activity, factors such as long-term structural stability, synthesis scalability, compositional control, and economic feasibility must be carefully evaluated. In addition, the performance of many reported catalysts under industrially relevant current densities and prolonged operating conditions remains insufficiently explored, limiting the assessment of their true technological potential.

From a fundamental perspective, the challenges associated with high-entropy catalysts can broadly be categorized into intrinsic and synthesis-dependent limitations. Intrinsic limitations originate from the inherent thermodynamic and kinetic complexity of multicomponent systems. These include heterogeneous atomic environments, fluctuating adsorption energetics, and the difficulty of identifying well defined catalytic active sites across diverse local configurations. Such intrinsic characteristics, while often responsible for the unique catalytic properties of high-entropy materials, can complicate the establishment of clear structure–activity relationships and hinder reliable prediction of catalytic behavior across vast compositional spaces. In contrast, synthesis-dependent limitations arise from practical fabrication challenges, including achieving homogeneous elemental distribution, controlling nanostructure morphology, preventing phase segregation during synthesis, and maintaining structural stability during long-term electrochemical operation.

**Figure 6:**
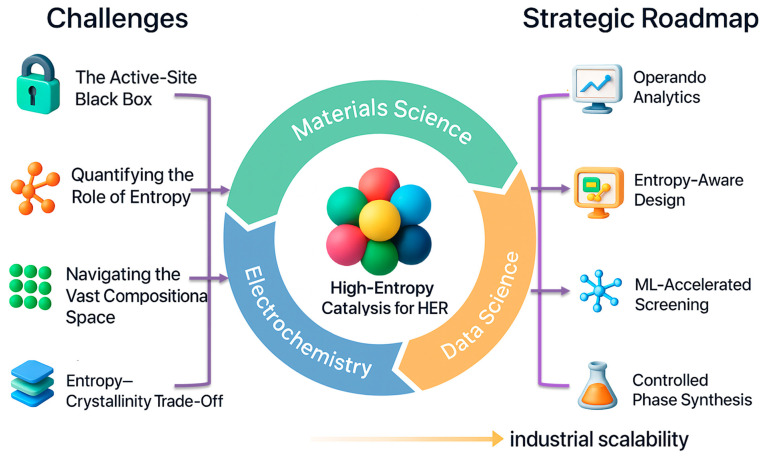
**Grand challenges and strategic roadmap for HECs toward the HER.** The illustration highlights a closed-loop framework uniting materials science, electrochemistry, and data science to accelerate the rational design of HECs. The left panel summarizes major challenges—including the active site black box, entropy quantification, compositional complexity, and entropy–crystallinity trade-off—while the right panel outlines strategic directions such as operando analytics, entropy-aware design, machine learning-accelerated screening, and controlled phase synthesis. This integrated, feedback-driven approach provides a mechanistic and scalable roadmap for advancing HECs from discovery to industrial deployment in HER systems.

As illustrated in the strategic framework of Figure 6, clearly distinguishing between intrinsic thermodynamic and kinetic constraints and synthesis-related fabrication challenges is essential for guiding rational catalyst design and improving the scalability of high-entropy electrocatalysts. Addressing these limitations will require coordinated advances in catalyst synthesis, mechanistic characterization, and predictive modeling.

In addition to the challenges outlined above, identifying the rate-determining step (r.d.s) in high-entropy catalysts remains a critical issue. Owing to the presence of multiple active sites with different adsorption energies, the HER of HECs may proceed through parallel pathways rather than a single dominant mechanism. As a result, it is often difficult to assign one specific r.d.s, since the Volmer, Heyrovsky, and Tafel steps may all contribute simultaneously depending on composition and operating conditions. Therefore, understanding and controlling the r.d.s in such multicomponent systems is essential for rational catalyst design and further performance optimization.

These considerations are therefore critical for evaluating the real technological potential of high-entropy catalysts. Accordingly, the following sections outline the key scientific and engineering challenges associated with high-entropy HER catalysts and discuss potential strategies to overcome these limitations, thereby accelerating their transition toward practical hydrogen production technologies.

### 4.1. The Active Site Black Box

Despite extensive reports of outstanding HER activity, the atomic origin of catalytic behavior in HEAs remains elusive. The compositional complexity of high-entropy catalysts generates a wide range of local atomic environments, which in turn produce a continuum of adsorption energies across the catalyst surface. While this diversity contributes to high catalytic activity, it also makes it difficult to identify the specific atomic motifs responsible for HER activity [14]. Without direct structure–activity correlation, performance metrics alone offer limited mechanistic insight [56].

**Figure 7:**
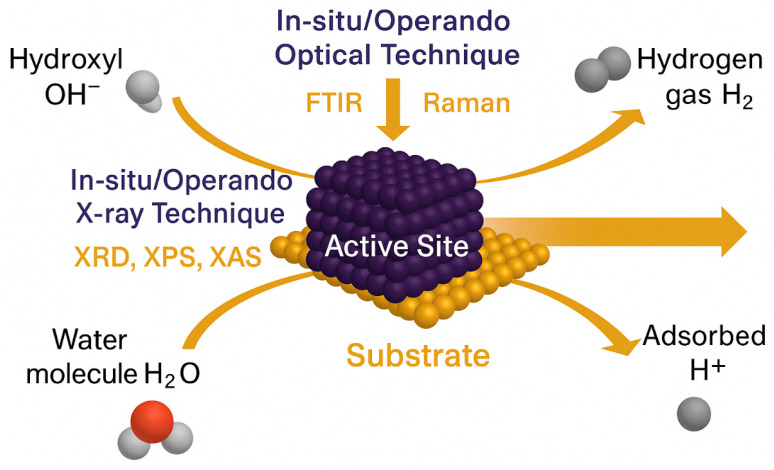
**Schematic illustration of in situ and operando characterization techniques applied to unravel active sites and intermediates during HER on high-entropy catalysts**. The violet nanoparticle cluster represents the active catalytic region supported on a gold substrate. Surrounding molecular species include water (H_2_O), hydroxyl (OH^−^), adsorbed (H^+^), and evolved hydrogen gas (H_2_), depicting key intermediates and products in the HER pathway. Optical probes such as Raman and FTIR provide vibrational insights, while X-ray-based techniques (XRD, XPS, XAS) offer structural and electronic information under working conditions. Together, these multimodal approaches enable real-time correlation between atomic-scale structure, electronic configuration, and catalytic activity.

To resolve this, operando atomic-scale analytics must become central to HEC research (Figure 7). Techniques such as Raman, FTIR, XRD, XPS, and XAS combined under multimodal, in situ configurations enable real-time tracking of surface intermediates and structural evolution [84]. When integrated with ab initio and DFT–ML data fusion, these tools can pinpoint element-specific coordination environments and reveal the true active sites. For instance, operando XAS studies on PdRhMoFeMn metallenes revealed that Mn facilitates OH adsorption and water dissociation, whereas Pd serves as the primary hydrogen-binding site [17,85,86].

Extending this approach, next-generation multimodal probes—including electrochemical X-ray photoelectron spectroscopy (EC-XPS), liquid-cell TEM, and nano-spot XAS coupled with photoelectron-emission microscopy (PEEM)—will allow spatiotemporal mapping of active site transformations [87]. Embedding these tools within iterative design loops—where synthesis, testing, and DFT feedback continuously refine alloy compositions—creates a systematic pathway toward rational catalyst engineering rather than empirical discovery.

### 4.2. Quantifying the Role of Entropy

Configurational entropy plays an important role in stabilizing multicomponent solid-solution structures in high-entropy catalysts and can indirectly influence the adsorption thermodynamics of intermediates such as H* and OH*. Yet most computational studies evaluate only enthalpic contributions (Δ*H*) at 0 K, neglecting vibrational and Δ*S_mix_*. These omissions can distort calculated free energies ΔG_H*_ by tens of meV, misrepresenting catalytic trends [88]. To correct this, entropy-resolved computation and calorimetry must become standard. Incorporating vibrational entropy through frequency analyses and Monte Carlo configurational sampling will yield more realistic ΔG_H*_ predictions. Experimentally, operando microcalorimetry and temperature-dependent kinetic studies can isolate enthalpic vs. entropic components, validating theoretical models [89].

Bridging simulation and experiment requires closed computational–experimental feedback loops that continuously refine thermodynamic parameters [90]. Combining DFT energetics, calorimetry, and operando spectroscopy (XAS, Raman) will create a self-correcting pathway that refines thermodynamic parameters iteratively. Within this loop, targeted compositional tuning—guided by DFT ΔG_H*_ analyses and verified by batch electrochemical testing—acts as a practical synthesis-to-performance validation step. In this context, entropy evolves from a passive stabilizer into an active design lever, enabling HECs optimized for both durability and activity.

### 4.3. Navigating the Vast Compositional Space

The combinatorial complexity of HEAs—spanning millions of quinary and senary formulations—poses both an opportunity and a bottleneck for catalytic discovery [91,92]. While existing empirical metrics such as valence-electron concentration and mixing enthalpy can describe bulk stability, they fail to predict catalytic performance at the active sites. To address this gap, the field must transition from empirical trial-and-error to data-driven, multi-objective optimization frameworks that integrate theory, computation, and experiment. In this direction, machine learning-guided high-throughput screening frameworks trained on ab initio and experimental datasets have emerged as powerful tools to navigate vast compositional landscapes. Algorithms such as graph neural networks and Bayesian optimization can efficiently predict hydrogen adsorption free energies (ΔG_H*_) and kinetic barriers while simultaneously balancing trade-offs between phase stability and catalytic performance [93]. To ensure thermodynamic feasibility, CALPHAD modeling can be incorporated to verify that the predicted alloys remain single-phase and stable under realistic synthesis conditions [49].

**Figure 8:**
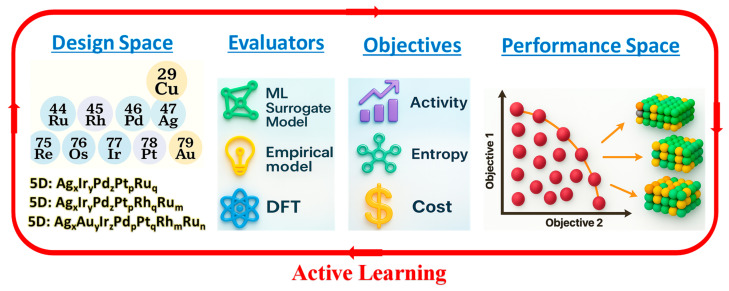
**Schematic illustration of a diversity-guided multi-objective active learning framework for HEC discovery.** The workflow integrates design space, evaluators, and objectives within a feedback-driven performance space loop. Diverse multimetallic combinations are generated within the design space; their catalytic performance is predicted via evaluators such as ML surrogate models, empirical correlations, and DFT computations. Multiple objectives—including activity, entropy, and cost—are simultaneously optimized to identify Pareto-efficient candidates. The iterative active learning cycle enables continuous refinement of the model toward efficient exploration and optimization of complex HEC systems for HER [14].

As illustrated in Figure 8, this diversity-guided multi-objective active learning framework embodies a closed-loop approach that unites materials design, computational evaluation, and experimental validation. The workflow begins with the design space, where diverse multi-elemental combinations (e.g., quinary or senary alloys such as Ag_*X*_Ir_*γ*_PdzPtyRu*_q_*) are generated based on configurational entropy principles. These compositions are then assessed by evaluators—including machine learning surrogate models, empirical thermodynamic models, and density functional theory (DFT) calculations—to predict key physicochemical parameters [14].

This iterative active learning cycle ensures that insights gained from experimental feedback are continuously fed into the predictive models, refining their accuracy and guiding the next round of material design. In practice, this active learning cycle seamlessly integrates computational prediction with tangible experimental workflow. The process begins with the synthesis of candidate alloys, often employing scalable methods like arc-melting, ball-milling, phosphorization or de-alloying to achieve the desired porous, single-phase HEC structures. Rigorous characterization using techniques such as TEM, HAADF-STEM/EDS, and XRD then verifies the compositional uniformity and phase purity, ensuring the fidelity of the crucial performance data (activity, kinetics, and short-term stability) which serves as the ground truth for the computational models. This experimental feedback is then used to retrain the predictive engines, such as by refining the DFT-calculated ΔG_H*_ values for the most promising elemental motifs. This iterative refinement, where insights from each experimental stage continually enhance the predictive power of the next design iteration, is the engine that drives the accelerated discovery of optimal HEC formulations. Collectively, this data-centric closed-loop framework accelerates the “discovery-to-deployment” continuum for high-entropy electrocatalysts by uniting materials science, electrochemistry, and data science within a self-improving feedback cycle.

### 4.4. Entropy–Crystallinity Trade-Off

Whether a HEA is crystalline or amorphous crucially affects its catalytic landscape, yet mechanistic connection is poorly resolved [94,95]. Crystalline structures offer electronic order and predictable motifs, whereas amorphous variants provide defect-rich surfaces with high densities of catalytic sites arising from their disordered atomic structures. Composition-controlled comparative synthesis is therefore essential. Producing identical alloys in crystalline and amorphous forms (via annealing or rapid quenching) and analyzing them using operando XRD, TEM, and Raman spectroscopy will illuminate how structural order influences kinetics. Parallel molecular dynamics and DFT simulations on amorphous surfaces can quantify how entropy reshapes ΔG_H*_ landscapes.

To operationalize this understanding, synthesis workflows should aim to achieve sub-5 nm ligament sizes with clearly resolved amorphous–crystalline domains, verified by HAADF-STEM and porosity mapping. Such precise control bridges fundamental understanding with manufacturable design, allowing targeted optimization of activity–stability trade-offs.

### 4.5. From Discovery to Industrial Scalability

Even with optimized compositions, synthesis scalability remains a critical challenge. Traditional small-batch methods (arc melting, sputtering, carbothermal shock) produce high-performance materials but suffer from poor reproducibility and phase segregation [96,97]. To enable industrial translation, green, continuous-flow, and low-temperature synthesis routes must be prioritized [98].

Techniques such as microwave-assisted alloying or electric field-driven sintering can deliver uniform nanoparticles with precise phase control. Automated systems—integrating precursor-purity monitoring, atmosphere regulation, and real-time compositional feedback—ensure reproducibility across laboratories. Combined with high-throughput pilot testing (≥500 mA cm^−2^), these methods bridge the gap between discovery and deployment [99,100,101]. Here, predictive design meets scalable manufacturing: computationally guided compositions are synthesized in parallel, rapidly screened, and refined through electrochemistry + DFT validation. The outcome is a validated, reproducible framework for entropy-engineered catalysts, directly translatable to industrial modules [102,103,104,105].

It should also be recognized that catalyst activity evaluated at laboratory benchmark conditions, such as η at 10 mA cm^−2^, does not fully reflect the requirements of practical water electrolyzers, which commonly operate at current densities exceeding 1 A cm^−2^. Under these conditions, additional factors including bubble nucleation and detachment, gas–liquid hydrodynamics, mass-transfer limitations, ohmic losses, local pH gradients, and thermal management become increasingly important. Excessive bubble coverage can block active sites and increase interfacial resistance, thereby diminishing the apparent catalytic advantages observed under low-current testing. Therefore, future development of high-entropy catalysts should increasingly emphasize high-current-density evaluation, integrated electrode design, and long-term durability under industrially relevant operating environments.

### 4.6. Ensuring Long-Term Stability

Although HEAs exhibit intrinsic thermodynamic stability due to sluggish diffusion and Δ*S_mix_*, their long-term behavior under operational stress remains insufficiently explored [106,107]. Under high current densities (≥500 mA cm^−2^), intense bubble evolution and Joule heating can induce surface reconstruction, compositional leaching, and interfacial delamination [108,109]. To address these degradation modes, operando endurance testing exceeding beyond 1000 h should integrate synchrotron XAS, TEM, and infrared imaging to track morphological and chemical evolution in real time. Complementary surface-engineering strategies—such as self-supported nanoarrays, covalently bonded monolithic electrodes, and superaerophobic textures—can mitigate bubble accumulation and delamination.

Beyond intrinsic stability, chemical durability in harsh electrolytes (acidic, alkaline, seawater) remains under-investigated. Operando ICP-MS, EC-TEM, and XAS can quantify element dissolution and oxide formation during operation, providing direct insight into corrosion mechanisms [48,106,110]. Comparative studies across different electrolytes will identify composition–stability relationships and enable the design of chloride-tolerant HEAs capable of long-term operation in real seawater environments [111].

### 4.7. Achieving Economic and Environmental Sustainability

While HEAs promise to reduce noble metal dependency, many high-performing formulations still rely on expensive Pt or Pd, elevating material costs [112]. Transitioning to non-precious-metal systems—incorporating Fe, Co, Ni, Mo, and Cr—can deliver competitive catalytic activity when guided by ML-optimized doping strategies that stabilize surface states and suppress corrosion. Rapid synthesis techniques such as carbothermal shock or top-down de-alloying of Al-based precursors can yield nanoporous architectures with ultra-low η and high durability [53,113,114,115]. Furthermore, low-temperature sol–gel, hydrothermal, and mechanochemical methods will significantly reduce energy input. Integrating techno-economic analysis (TEA) and life-cycle assessment (LCA) during early design phases provides quantitative metrics for cost, emissions, and resource efficiency, ensuring alignment with global green hydrogen targets. Such frameworks will distinguish genuinely sustainable catalysts from merely high-performing ones.

### 4.8. Summary and Outlook

In summary, HECs offer an unprecedented combination of compositional diversity, tunable electronic landscapes, and structurally stable multicomponent architectures capable of transforming hydrogen evolution catalysis. Realizing this potential, however, demands a coordinated, multipronged roadmap that systematically links mechanistic insight to industrial application. The strategic trajectory forward requires resolving the mechanistic puzzle of active sites through advanced operando analytics, while simultaneously embedding entropy as a quantitative design variable in thermodynamic–experimental feedback loops. This must be coupled with implementing ML-accelerated, high-throughput optimization cycles to navigate the vast compositional space and converge on stable, high-performance alloys. Finally, these scientific advances must be grounded through the development of green, reproducible synthesis routes, validated at pilot scale and guided by rigorous TEA and LCA metrics. By integrating these directions into a unified workflow, the scientific community can accelerate the journey of HECs from fundamental discovery to industrial impact, ultimately delivering the durable, cost-effective, and sustainable catalysts for a global hydrogen economy.

## 5. Conclusions and Outlook

HECs are not merely an incremental improvement in HER electrocatalysis; they represent a fundamental paradigm shift. By leveraging a compositional diversity, synergistic multi-element interactions, and intrinsic structural disorder, HECs have shattered the design constraints of traditional materials. This has enabled the creation of vast, tunable compositional landscapes, leading to catalysts with precisely engineered active sites that rival or even surpass platinum. The remarkable experimental breakthroughs—achieving overpotentials as low as 10 mV and demonstrating exceptional stability at industrial-scale current densities—are a testament to this potential. The field is now rapidly moving beyond precious metals, proving that cost-effective, earth-abundant HECs can spearhead the future of sustainable hydrogen production.

However, translating this immense potential into tangible technology requires a deeply integrated, interdisciplinary approach. The path forward is not linear but cyclical, demanding a powerful closed-loop workflow that unites materials science, electrochemistry, and data science. In this collaborative framework, computational predictions from DFT and machine learning guide the rational synthesis of novel HECs. Advanced operando characterization then provides crucial, real-world feedback on their structure–activity relationships under operational conditions. This experimental data, in turn, is used to retrain and refine the predictive models, accelerating the discovery cycle far beyond what trial-and-error methods could ever achieve. It is this fusion of disciplines that will unlock the rational design of catalysts that are simultaneously active, durable, and economically viable.

Ultimately, the future vision for HECs is one of synergistic innovation. By uniting materials discovery, mechanistic insight, and intelligent design, we can accelerate the development of catalysts engineered not just for peak performance, but for holistic sustainability—validated by rigorous life-cycle and techno-economic assessments. HECs, with their diverse atomic environments, entropy-stabilized structures, and data-driven design workflows, offer a powerful and scalable route to achieving this goal. They are not just a promising class of materials; they are a cornerstone of the emerging clean energy revolution, poised to redefine the landscape of green hydrogen technology for generations to come.

## Figures and Tables

**Table 1:** Conceptual comparison of high-entropy catalysts (HECs) and traditional catalysts. The values listed are representative ranges from the literature and are not obtained under identical experimental conditions.

Feature	Traditional Catalysts (Pt, Pd, etc.)	HECs	Representative References
Configurational entropy	Δ*S_mix_* = 0	High Δ*S_mix_*; stabilizes single-phase solid solutions	[4,10,11,12]
Phase/stability	Prone to sintering and dissolution	Sluggish diffusion and high-entropy suppress phase breakdown	[11,12]
Active site diversity	Uniform single-site behavior	Broad spectrum of active sites with cooperative catalytic roles	[9,12]
HER performance ($\eta_{10}$)	Pt/C ≈ 17–60 mV (acidic media)	HEAs/HEOs reported as low as ≈9–23 mV	[13,15,16,17]
Durability	Rapid degradation under cycling	>100 h stability in harsh electrolyte environments	[16,17]
Cost & scalability	Noble metal-heavy; high cost	Reduced noble metal content; scalable synthetic strategies emerging	[13,49]

**Table 2:** Synthesis methods, key features, advantages, limitations, and scalability of high-entropy catalysts. Scalability considerations are summarized based on the recent literature [6].

Synthesis Method	Key Features	Advantages	Challenges/Limitations	Scalability	Relative Cost/Industrial Benchmarks	Representative Examples
Mechanical Alloying (MA)	Solid-state powder processing involving repeated cold welding and fracturing under high-energy milling.	Scalable, cost-effective; promotes uniform atomic mixing; suitable for bulk HEAs (FCC, BCC, amorphous).	Risk of contamination from milling media; strict control of milling time, atmosphere, and speed needed; limited morphology control.	High (industrial-scale feasible)	Moderate; competitive if noble metal-free compositions are used	FeNiCoCuMo, CoCrFeNi, (FeCoNiCuAl_2_Mn) HEAs and high-entropy oxides (HEOs) [52,58].
Carbothermal Shock (CTS)	Ultrafast flash heating (~2000 K for <50 ms) followed by rapid quenching.	Produces nanoscale HEA particles with high phase uniformity and minimal segregation; tunable composition.	High-energy requirement; precise thermal control; limited scalability; substrate compatibility critical.	Low–moderate (energy-intensive, limited scale)	High process cost; less competitive than Ni-based catalysts currently	PtPdRhRuCe/C, FeCoNiCuMo–O HEOs, PdFeCoNiMo [52].
Solvothermal/Hydrothermal Synthesis	Solution-phase growth under high T/P in sealed autoclaves.	Enables morphology and size control under mild conditions; scalable for nanostructures; compatible with oxides/sulfides.	Long reaction times; solvent-dependent phase selectivity; atomic-level mixing less controlled.	Moderate–high (scalable with reactors)	Moderate; composition-dependent precursor cost	NiCoMnFeCrO*_x_*, FeNiMoS_2_, CoNiMoP [59].
Arc/Plasma/Spark Discharge	Rapid melting + solidification through localized plasma or arc discharge.	Produces dense, homogeneous alloys; favors uniform phase formation; suitable for conductive supports.	High-temperature process; complex equipment; poor control of nanoparticle size; costly.	Low (lab-scale, costly)	High capital and energy cost	CoCrFeNiMn, NiFeCoCu-based HEAs [52].
Laser Ablation/Laser Pyrolysis	Laser pulse-induced evaporation/condensation of targets in gas or liquid medium.	Yields ultra-pure nanoparticles; tunable by laser fluence and atmosphere; contamination-free.	Low yield; specialized optics; limited scalability.	Low (limited yield)	High cost; limited industrial competitiveness	FeCoNiCuMo nanoparticles, PtPdNiCo HEA clusters [52].
Flash Pyrolysis/Rapid Thermal Processing	Short-duration heating (hundreds ms) of precursors in controlled atmospheres.	Rapid nucleation; forms metastable HECs; potentially scalable.	Precise ramp-rate control required; risk of incomplete reaction/agglomeration.	Moderate–high (continuous processing possible)	Moderate; promising if continuous large-scale processing is achieved	FeCoNiCrMoO_x_, CuNiCoMnFe sulfides [52].
Electric Field-Assisted Sintering (Spark Plasma Sintering)	Uses pulsed electric current and pressure for rapid powder consolidation.	Very short sintering time; fine-grained microstructure; enhanced diffusion and densification.	Expensive setup; limited to conductive materials; possible interfacial oxidation.	Moderate (limited by equipment size)	Moderate–high equipment cost	FeCoNiCrMo, TiZrNbMoTa [52].

**Table 3:** Comparative overview of different HEC classes for HER.

Feature	HEAs	HEOs/HESs	HEPs	MXenes
**Representative examples**	PtPdRhRuCu [46]; FeCoNiCuAl_2_Mn [63]; CoCrFeNi [64]	(FeMnNiCoMo)S_2_ [70]; CuCoNiMnCrS_x_ [38]; (ZnCoMnFeAlMg)_9_ S_8_ [71]; TiHfZrNbTaO_11_ [72]	La_2_(CoNiMgZnNaLi)RuO_6_ [39]; La(CrMnFeCoNi)O_3_ [77]; Sr-doped La_0.3_Sr_0.7_(CrMnFeCoNi)O_3_ [78]	TiVNbMoC_3_ [80]; TiVNbMoAlC_3_ [81]; MXene hybrids like Ti_3_C_2_–TiO_2_ [83]
**Key mechanisms**	Multi-metal synergy; charge redistribution (e.g., Fe/Co donating to Pt) [66]; lattice distortion suppresses segregation [69]	Self-reconstruction into MOOH [74]; Cu^+^ “soft acid–hard acid” effect; nanostructure multiplicity; entropy-driven bandgap tuning [76]	Alkali-induced super-exchange facilitating e^−^ transfer, e.g., filling (~1.2) enables continuum of sites; defect engineering via A/B site disorder [79]	Expanded interlayer spacing (1.5–1.8 nm); ensemble effect tuning ΔG_H*_ near thermoneutral; termination control (−O, −OH, −F) [82]
**HER performance (** $\eta_{10}$ **)**	As low as 9.7–23 mV; broad pH stability (acid + alkaline)	Onset ~0.25 V at 100 mA cm^−2^; $\eta_{10}$ ≈ 170 mV alkaline; photocatalytic HER under visible light	$\eta_{10}$ ≈ 40.7 mV (alkali-activated); long-term durability (>200 h)	ΔG_H*_ ≈ −0.41 eV (O-terminated); high surface area (~28 m^2^ g^−1^) enables robust HER
**Durability**	>100 h stability; up to 10,000 cycles without degradation	Stable at 1 A cm^−2^ for >100 h; Ru sulfides preserve activity via oxyhydroxide transformation	>200 h stability in alkaline HER [57]	Prevent restacking via crumpled morphologies; stable hybrids with TiO_2_
**Advantages**	Platinum-like activity, multi-pH universality, scalable fabrication (pyrolysis, dealloying)	Noble metal-free, bifunctionality (HER/OER), scalable synthesis	Durable oxide frameworks, tunable electronic structure, stable water splitting	2D conductivity, termination tunability, versatile for hybrids/photocatalysis
**Challenges**	Element segregation at scale; synthesis cost (noble content)	Phase stability, reconstruction control under long-term cycling	Limited conductivity; early-stage development	Restacking tendency; synthesis complexity

## Data Availability

The data are available from the corresponding author on reasonable request.
